# Prevalence of common risk factors of major noncommunicable diseases among sexual and gender minorities in Kathmandu valley, Nepal

**DOI:** 10.1097/MD.0000000000037746

**Published:** 2024-04-05

**Authors:** Bikram Poudel, Kiran Paudel, Bikram Adhikari, Rajan Paudel, Sandesh Bhusal, Nabin Adhikari, Tara Ballav Adhikari, Vishnu Prasad Sapkota, Roman Shrestha

**Affiliations:** a Central Department of Public Health, Institute of Medicine, Tribhuvan University, Kathmandu, Nepal; b Dhulikhel Hospital Kathmandu University Hospital, Dhulikhel, Nepal; c Nepal Health Frontiers, Kathmandu, Nepal; d Department of Allied Health Sciences, University of Connecticut, Storrs, Connecticut; e Community-Based Management of Non-communicable Diseases in Nepal Project, Nepal Development Society, Bharatpur, Nepal; f Section for Global Health, Department of Public Health, Aarhus University, Aarhus, Denmark; g Department of Economics, Tribhuvan University, Kathmandu, Nepal; h Section of Infectious, Department of Internal Medicine, Yale School of Medicine, New Haven, CT.

**Keywords:** Nepal, noncommunicable diseases, risk factors, sexual and gender minorities

## Abstract

Four noncommunicable diseases (NCDs): cardiovascular diseases, cancers, chronic respiratory diseases, and diabetes, account for 71% of global deaths. However, little is known about the NCDs risk profile of sexual and gender minorities (SGMs). This study aimed to determine the prevalence of NCDs risk factors among the SGMs of Kathmandu valley, Nepal. A cross-sectional study was conducted among SGMs in the Kathmandu valley, Nepal. We recruited 140 participants using the snowball sampling method. A face-to-face interview was done using a structured questionnaire adapted from World Health Organization Step Wise Approach to Surveillance (STEPS instruments V2.2 2019) along with blood pressure and anthropometric measurements. Data were analyzed using Statistical Package for Social Science (SPSS.v20). More than two-thirds of the participants, 96 (68.6%), had co-occurrence of NCDs risk factors. The prevalence of insufficient fruits and vegetables consumption, current smoking, harmful alcohol consumption, overweight/obesity, and hypertension were 95.7%, 40.0%, 32.9%, 28.5%, and 28.6%, respectively. There was a significant association between hypertension, harmful alcohol consumption, and overweight/obesity with the participants’ age, employment status, and marital status, respectively. Study findings indicated a higher prevalence of NCDs risk factors among SGMs. National-level NCDs surveillance, policy planning, prevention, and targeted health interventions should prioritize the SGMs.

## 1. Introduction

Noncommunicable diseases (NCDs) are an increasingly significant contributor to the global burden of diseases. In 2018, the World Health Organization (WHO) estimated that 71% of total global deaths and 75% of all premature adult deaths in those aged 30 to 69 years were caused by NCDs.^[1]^ Although traditionally labeled as the misery of high-income countries, an alarming 78% of all NCDs-related deaths and 85% of all premature adult NCDs-related deaths occurred in low and middle-income countries.^[2]^ The estimated economic burden due to NCDs for the 2010 to 2030 period is predicted to be 47 trillion US dollars, approximately 75% of the global Gross Domestic Product.^[3]^ In Nepal, two-thirds of total deaths and 59% of total Disability Adjusted Life Years were due to NCDs in 2017.^[4]^

Four major NCDs: cardiovascular diseases, cancers, chronic respiratory diseases, and diabetes, accounted for 60% of global deaths.^[1]^ In Nepal, these 4 NCDs caused 53% of total mortality.^[5]^ Modifiable behaviors: tobacco use, alcohol consumption, unhealthy diet, physical inactivity, and metabolic changes: overweight/obesity, raised blood pressure (BP), raised blood glucose, and raised blood cholesterol increase the risk of NCDs.^[6]^ The 2019 Step Wise Approach to Surveillance (STEPS) Survey in Nepal indicates a higher prevalence of NCD risk factors such as insufficient fruits and vegetables consumption (97%), raised BP (24.5%), overweight/obesity (24.3%), and smoking (17%) in the general Nepalese population. Early identification and prevention of these risk factors are vital in reducing the burden of NCDs.^[7]^

Sexual and gender minorities (SGMs) is an umbrella term that “encompasses lesbian, gay, bisexual and transgender (LGBT) people and as well as those whose sexual orientation or gender identity varies and may not self-identify as LGBT (e.g., queer, questioning, 2-spirit, asexual, men who have sex with men [MSM], gender-variant), or those who have a specific medical condition affecting reproductive development [DSD], who sometimes identify as intersex).”^[8]^ Limited available evidence suggests that SGMs are exposed to higher rates of modifiable risk factors for NCDs, such as elevated tobacco use and alcohol consumption than their heterosexual counterparts^[9,10]^ due to higher stress and mental health issues such as anxiety and depression that arise from their daily battles against discrimination, isolation, and rejection.^[11,12]^ The sustainable development goals, in particular goal number 3.4, targets to reduce the premature mortality from NCDs by one-third by 2030.^[13]^ The ambitious goal can only be achieved if the NCDs’ burden on the vulnerable section of the community like SGMs is identified and interventions are targeted toward them.

Nepal’s multisectoral action plan for the prevention and control of NCDs (2021–2025) targets a 25% reduction in premature mortality from NCDs. This action plan includes a periodic NCD STEPS survey as one of the key activities to track NCDs’ prevention and control progress within the country.^[14]^ These surveys have largely overlooked the SGMs, and thus, little is known about the NCDs risk profile of this population in Nepal. Therefore, this study aimed to determine the prevalence of risk factors of major NCDs among the SGMs of Kathmandu valley, Nepal.

## 2. Methods

### 2.1. Study design, setting, and population

A cross-sectional study was conducted among SGMs such as lesbian, gay, bisexual, and transgender living in the Kathmandu valley of Nepal from October to November 2019. Administratively, Kathmandu valley consists of 3 districts: Bhaktapur, Kathmandu, and Lalitpur. It is the most populated region of the country, characteristically resided by diverse population subgroups, including the SGMs population. Inclusion criteria included individuals aged 18 years and above who identified themselves as members of the SGM group and individuals living in the Kathmandu valley for at least 6 months at the time of data collection. The participants who did not provide consent were excluded from the study.

### 2.2. Sample size calculation and sampling

The estimated sample size was 140. It was calculated using Cochrane formulae (n = z²pq/d²) assuming 30% prevalence (p) of tobacco use,^[15]^ 8% allowable error (d), 95% confidence interval (CI), and 10% nonresponse rate. To ensure representative and maximum recruitment of this hidden population, snowball sampling, a nonprobability sampling technique, was applied.^[16]^ The sampling process started by purposively recruiting 4 primary seeds having diverse age, sexual orientations, and ethnic and occupational backgrounds with the help of Blue Diamond Society Nepal, a major nongovernmental organization that provides service to SGM in Nepal. All SGMs encountered through snowball sampling who met the eligibility criteria for our study were enrolled until the target sample size was met.

### 2.3. Data collection

We conducted face-to-face interviews using standardized questionnaires from the WHO STEPwise approach to NCD risk factor surveillance (STEPS) instrument version 2.2.^[17]^ The tool was previously translated into the Nepali language and adopted in the nationwide survey in Nepal**.**^[15]^ The tool prescribes 3 steps for measuring NCDs risk factors but for this study; here we included 2 steps: STEP I (sociodemographic information, NCDs risk factors information- physical activities, tobacco and alcohol consumption, fruit and vegetable intake); and STEP II (anthropometric measurement- height and weight and BP measurement-Systolic and diastolic BP). The measures also included questions related to a history of raised BP, glucose, and cholesterol.^[17]^ The principal investigator conducted all the face-to-face interviews and anthropometric and BP measurements.

### 2.4. Measurements

#### 2.4.1. Sociodemographic information.

It included age (in years), religion (Hindu, non-Hindu), ethnicity (Janajati, Brahmin/Chhetri, others), education level (primary level or below, secondary level, higher secondary level, bachelor or above), sexual orientation (gay, lesbian, bisexual, and transgender), marital status (never married, married, others), employment status (employed and unemployed), occupation (service, sex work, self-employed, labor), and current living status (with family, without family).

#### 2.4.2. NCDs risk factors information.

NCDs risk factors included current smoking, harmful alcohol consumption, insufficient fruits and vegetables intake, insufficient physical activity, overweight and obesity, and raised BP. Current smoking was considered if the participants had smoked in the past 30 days preceding the study. Consumption of ≥60 grams of pure alcohol on an average per day was considered harmful use of alcohol, and such drinkers were called binge drinkers. Consumption of <5 standard servings (≥400 grams) of fruits and vegetables on an average day was considered as insufficient fruits and vegetables intake. Participants who participated in less than the equivalent of 150 minutes of moderate-intensity (600 METs) physical activity per week were considered to have insufficient physical activity.^[18]^

#### 2.4.3. Co-occurrence of risk factors.

Participants were considered to have co-occurrence of risk factors if they were exposed to 2 or more risk factors of NCDs mentioned above.^[15]^

#### 2.4.4. Anthropometric measurement.

We measured the weight and height of the participants using the SAMSO digital weighing machine and nonelastic measuring tape, respectively. All measurements were conducted following the WHO STEPS guide to physical measurement strictly.^[18]^ We calculated body mass index (BMI) from height and weight using the formula: BMI = (weight in kg)/(height in meter).^[2,19]^ Participants with a BMI score in the range of 25.0 to 29.9 were considered overweight, and those with a BMI score of ≥30 were considered obese.^[18]^

#### 2.4.5. Blood pressure measurement.

We measured BP using a digital sphygmomanometer (OMRON HEM-7120 Automatic Blood Pressure Monitor; with indicated accuracy:  *±*3 mm Hg). We strictly followed the WHO STEPS guide to measure BP. Participants were considered to have raised BP if the average of the last 2 measurements of systolic BP was ≥140 mm HG and/or the average diastolic BP was ≥90 mm Hg and/or if they reported being previously diagnosed hypertensive patients or taking antihypertensive drugs.^[18]^

### 2.5. Statistical analysis

Data were compiled, edited, and checked for consistency, and processed through EpiData V.3.1 before exporting to the IBM SPSS version 20 for statistical analysis. Categorical variables were presented as frequency and percentage, and parametric numerical variables as mean and standard deviation. Univariate logistic regression analysis was used to determine the association of common risk factors of NCDs with sociodemographic characteristics. A *P* value of <.05 was considered statistically significant.

### 2.6. Ethical considerations

Ethical approval for the study was obtained from the Nepal Health Research Council (Reference number: 702/2019). Written permission for data collection was taken from Blue Diamond Society Nepal. Written informed consent was obtained from the participants before enrolling them in the study. The purpose of the study, procedures involved in the data collection, and potential benefits and harm of the study were clearly explained to the participants. We maintained confidentiality by keeping participants’ information on a password-protected computer and assured the voluntary participation of each participant.

## 3. Results

### 3.1. Sociodemographic characteristics of the participants

We approached 154 SGMs population aged 18 years and above, of which 140 responded to our study. Fourteen participants declined to participate or left the interview in the middle due to time limitations. More than half of the participants (59.3%) were aged between 18 and 29 years, with a mean age (±standard deviation) of 29.3** ± **9.3 years. Out of 140 participants, 42.9% were transgender, 40.0% were gay, 11.5% were lesbian, and 6.4% were bisexual. Among the 98 (70.0%) participants who were employed, nearly two third (61.1%) of them were involved in service (private job), followed by commercial sex work (21.4%) as shown in Table 1.

**Table 1:** Sociodemographic characteristics of the participants (N = 140).

Characteristics		Number, (%)
Age (in years)	18–29	83 (59.3)
	30–34	45 (32.1)
	45–69	12 (8.6)
	Mean ± SD	29.3 ± 9.3
Religion	Hindu	97 (69.3)
	Non-Hindu	43 (30.7)
Ethnicity	Janajati	82 (58.6)
	Brahmin/Chhetri	48 (34.3)
	Others (Dalit, Muslim, and Madhesi)	10 (7.1)
Education level	Primary level or below	16 (11.4)
	Secondary level	51 (36.4)
	Higher secondary level	47 (33.6)
	Bachelor or above	26 (18.6)
Sexual orientation	Transgender	59 (42.1)
	Gay	56 (40.0)
	Lesbian	16 (11.5)
	Bisexual	9 (6.4)
Marital status	Never married	95 (67.8)
	Married	39 (27.9)
	Others (divorce, living together)	6 (4.3)
Employment status	Employed	98 (70.0)
	Unemployed	42 (30.0)
Occupation (n = 98)	Service (private sector)	60 (61.1)
	Sex worker	21 (21.4)
	Self-employed (business, freelancing)	10 (10.2)
	Labor	7 (7.2)
Current living status	With family	95 (68.0)
	Without family	45 (32.0)

SD = standard deviation.

### 3.2. Prevalence of NCDs risk factors

**Figure 1.**
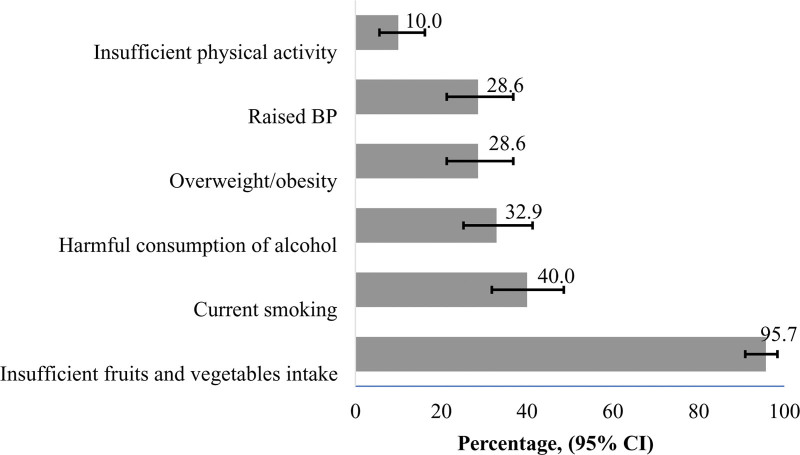
Prevalence of NCD risk factors among participants (N = 140). BP = blood pressure, CI = confidence interval, NCD = non-communicable disease.

The majority of the participants (95.7%) consumed less than 5 servings of fruits and vegetables in a day. The prevalence of current smoking among the total participants was 40.0%. Harmful consumption of alcohol was observed in nearly one-third (32.9%) of the participants. Nearly one-third (28.6%) of the participants were either overweight or obese. Similarly, the prevalence of raised BP, including those on medication for hypertension, was 28.6%. One in 10 participants had insufficient physical activity (Fig. 1).

### 3.3. Co-occurrence of risk factors among SGMs

**Figure 2.**
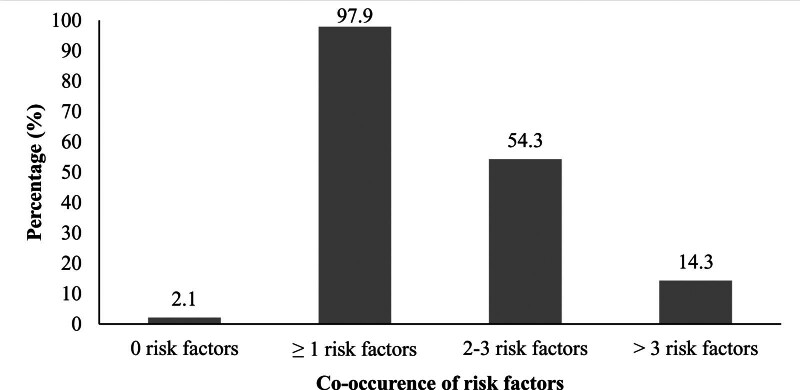
Co-occurrence of risk factors of NCDs among SGMs (N = 140). NCDs = non-communicable diseases.

Almost all participants (97.9%) had at least one of the 6 risk factors present. More than half (54.3%) of the participants had 2 to 3 risk factors present. Co-occurrence of more than 3 risk factors was found in 14.3% of the participants (Fig. 2).

### 3.4. Factors associated with the NCDs risk factors

A statistically significant association (*P* = .001) was found between the harmful consumption of alcohol and the employment status of the participants. The odds of harmful consumption of alcohol among employed participants were 6 times higher (odds ratio [OR]: 6.0; 95% CI: 1.9–19.2) than the unemployed participants.

Similarly, a significant association was found between the participants’ marital status (*P* = .001) and overweight or obesity. Married participants were more likely to be overweight or obese as compared to unmarried participants (OR: 4.3; 95% CI: 1.9–9.5) (Table 2).

**Table 2:** Factors associated with the NCDs risk factors.

Variables	Current smoking					Harmful consumption of alcohol				Overweight/obesity			
	n (%)		OR (95% CI)	*P* value		n (%)	OR (95% CI)		*P* value	n (%)	OR (95% CI)		*P* value
Age group													
Below 30	32 (38.5)		1	.7		22 (26.5)	1		.05	20 (24.1)	1		.2
30 and above	24 (42.1)		1.2 (0.6–2.3)			24 (42.1)	2.5 (1.0–6.4)			20 (35.1)	1.7 (0.8–3.6)		
Employment status													
Unemployed	12 (28.6)		1	.7		5 (11.9)	1		.001*	8 (19.0)	1		.3
Employed	44 (44.9)		2.0 (0.9–4.4)			41 (41.8)	6.0 (1.9–19.2)			32 (32.7)	1.7 (0.8–3.6)		
Marital status													
Unmarried	40 (39.6)		1	.9		35 (34.7)	1		.5	20 (19.8)	1		.001*
Married	16 (41.0)		1.1 (0.5–2.3)			11 (28.2)	3.1 (0.8–12.3)			20 (51.3)	4.3 (1.9–9.5)		
Educational status													
Upto SLC	29 (43.3)		1	.5		21 (31.3)	1		.7	19 (28.4)	1		.9
Above SLC	27 (37.0)		0.8 (0.4–1.5)			25 (34.2)	0.6 (0.2–1.5)			21 (28.8)	1.0 (0.5–2.1)		
Current living status													
Without family	23 (51.1)		1	.06		18 (40.0)	1		.2	12 (26.7)	1		.7
With family	33 (34.7)		2.0 (1.0–4.0)			28 (29.5)	1.1 (0.4–2.8)			28 (29.5)	0.9 (0.4–1.9)		

Variables	Raised blood pressure					Insufficient physical activity				Insufficient fruits and vegetables intake			
	n (%)	OR (95% CI)			*P* value	n (%)		OR (95% CI)	*P* value	n (%)		OR (95% CI)	*P* value
Age group													
Below 30	13 (22.0)	1			.001*	10 (12.0)		1	.33	79 (95.2)		1	.7
30 and above	27 (51.9)	3.8 (1.7–8.7)				4 (7.0)		0.6 (0.2–1.9)		55 (96.5)		0.7 (0.1–4.1)	
Employment status													
Unemployed	8 (29.6)	1			.1	6 (14.3)		1	.3	39 (92.9)		1	.4
Employed	32 (38.1)	1.5 (0.6–3.7)				8 (8.2)		0.5 (0.2–1.6)		95 (96.9)		0.4 (0.8–2.1)	
Marital status													
Unmarried	25 (32.5)	1			.1	12 (11.9)		1	.2	98 (97.0)		1	.2
Married	15 (44.1)	1.6 (0.7–3.8)				2 (5.1)		0.4 (0.5–9.9)		36 (92.3)		2.7 (0.5–14.1)	
Educational status													
Under SLC	22 (43.1)	1			.3	7 (10.4)		1	.9	62 (92.5)		1	.07
SLC and above	18 (30.0)	0.6 (0.2–1.2)				7 (9.6)		0.9 (0.3–2.7)		72 (98.6)		0.2 (0.2–1.5)	
Current living status													
Without family	17 (44.7)	1			.9	7 (15.6)		1	.1	44 (97.8)		1	.4
With family	23 (31.5)	1.8 (0.8–3.9)				7 (7.4)		2.3 (0.9–7.1)		90 (94.7)		0.4 (0.1–3.6)	

CI = confidence interval, COR = crude odds ratio, SLC = School Leaving Certificate.

* *P*  < .05.

A significant association (*P* = .001) was found between raised BP and the age of the participants. Participants aged 30 and above were nearly 4 times (OR: 3.8; 95% CI: 1.7–8.7) more likely to have raised BP than those below 30 years of age.

Although a significant association was not found between employment, marital, current living status, and insufficient physical activity but participants who were unemployed, unmarried, and living without family were more likely to have insufficient physical activity than those who were employed, married, and living with the family, respectively (Table 2).

A significant association (*P* = .001) was found between raised BP and the age of the participants. Participants aged 30 and above were nearly 4 times (OR: 3.8; 95% CI: 1.7–8.7) more likely to have raised BP than those below 30 years of age.

Although a significant association was not found between employment, marital, current living status, and insufficient physical activity but participants who were unemployed, unmarried, and living without family were more likely to have insufficient physical activity than those who were employed, married, and living with the family, respectively (Table 2).

## 4. Discussion

Our study showed the higher prevalence of common risk factors of major NCDs among SGM in Nepal. More than two-thirds of the participants had co-occurrence of 2 or more than 2 risk factors of NCDs. The study showed a significant association between hypertension, harmful alcohol consumption, current smoking, and overweight/obesity with the participants’ age, employment status, sexual orientation, and marital status, respectively.

### 4.1. Current smoking and alcohol consumption

The prevalence of current smoking (40.0%) among the SGMs is double than that of the general population living in the region (18.8%), as reported by the STEPS survey 2019 of Nepal.^[7]^ This comparable difference in smoking prevalence among the SGMs and the general population is consistent with the findings reported by Tang et al, Dai, and Gruskin et al from the USA.^[10,20,21]^ A higher prevalence of smoking was also reported among transgender in Nepal (58.2%)^[22]^ and India (43.5%).^[23]^

Harmful alcohol consumption among the SGMs is alarmingly higher (32.9%) when compared with the general population (8.72%) in Bagmati province.^[7]^ Similar disparities in alcohol consumption between the SGMs and their heterosexual counterparts were reported in several studies.^[9,24,25]^ The prevalence of harmful consumption of alcohol reported in our study is greater than the study conducted among transgender (21%) in India.^[23]^ Although not clearly stated as harmful use, a higher prevalence of consumption of alcohol (68.5%) was reported among transgender in Nepal.^[22]^

Many psychosocial and structural risk factors may contribute to the higher prevalence of smoking and alcohol consumption.^[26]^ Social stressors associated with transgender identities, such as identity development and related conflicts, might lead to the burgeoned substance used as a coping mechanism. Discrimination, chronic level of harassment, violence, and mental health disorders can also influence substance use.^[27]^ Higher harmful consumption of alcohol among employed (41.8%) than unemployed participants (11.9%) reported in this study might be due to job-related stress^[28]^ and higher purchasing power^[29]^ among employed participants.

### 4.2. Insufficient fruits and vegetables consumption and physical inactivity

The prevalence of insufficient fruits and vegetables consumption among the SGMs found in this study is on par with that of the general population living in Bagmati province^[7]^ but higher than reported by Madhavan et al^[23]^ among transgender in India.

In this study, a low level of physical activity was seen in 10% of the participants. This is similar to the findings reported by the STEPS survey 2019 among the general population living in the Bagmati province^[7]^ but much lower than those reported by Madhavan et al^[23]^ among transgender in India.

There were no significant differences in insufficient fruits and vegetables consumption and physical activity with independent variables included in this study. So further study with including more variables would be helpful in findings the reasons for insufficient fruits and vegetables consumption.

### 4.3. Overweight and obesity

The proportions of overweight and obese participants (28.6%) in this study are lower in comparison to the general population living in the Bagmati province (42.8%), as reported in the STEPS survey 2019 of Nepal.^[7]^ A study from India among transgender reported a higher prevalence of overweight (16%) and obesity (34.5%).^[23]^ Also, a higher prevalence of overweight and obesity among lesbians was reported by Boehmer et al^[30]^ in the USA. We found a significant association between marriage and obesity; this might be because they might involve in hormone therapy after marriage. Kyinn et al^[31]^ showed that following 11 to 21 months of hormone therapy, weight gain ≥5 kg was seen among 21% of the transfeminine individuals and 30% of Tran’s masculine individuals.

### 4.4. Raised BP

The prevalence of raised BP among the SGM participants in this study (28.6%) is slightly higher than the findings reported by the STEPs Survey 2019 among the general population living in the Bagmati province (25.2%).^[7]^ Some studies from the USA also reported a higher prevalence of raised BP among SGMs compared to the general population.^[32]^ The higher prevalence of raised BP among SGMs might be due to a higher prevalence of behavioral risk factors such as binge drinking and current smoking coupled with increased stress due to common discrimination and stigmatization.

### 4.5. Co-occurrence of risk factors

Almost all SGMs participants (97.9%) in this study had at least one of the 6 risk factors present. This is slightly less than reported by the STEPS Survey 2014 among the general population (99.6%) in Nepal. More than two-thirds (68.6%) of the participants in our study had co-occurrence of 2 or more than 2 risk factors which is higher than the general population (58.6%) reported in the previous STEPS survey in Nepal.^[15]^

However, the study has some limitations which need to be acknowledged. First, most of the measurements were based on self-reports, which may have been prone to information and recall bias. Second, we could not include the STEP III (biochemical measurements) of the standard tool due to resource constraints. Third, a nonprobability sampling technique and a small sample size used in this study may not accurately reflect the study population. Lastly, due to resource limitations, we couldn’t include the STEP III of the standard tool, which might have identified other metabolic risk factors of NCDs among SGMs. Despite limitations, this study provides evidence on the NCD risk factors among SGMs in Nepal. To the best of our knowledge, this study is the first to assess the risk factors of NCDs among SGM in Nepal, which could be of interest to policymakers, the government of Nepal, and those involved in the welfare of the SGMs population. Implement comprehensive and culturally sensitive healthcare policies, including accessible and affordable NCD prevention and management programs tailored specifically for sexual minorities in low-resource country settings, while prioritizing community engagement, stigma reduction, and equitable access to care.

## 5. Conclusion

Our study shows a higher prevalence of current smoking, harmful alcohol consumption, insufficient fruits and vegetables intake, overweight/obesity, and raised BP among the SGMs of the Kathmandu valley. Also, a large proportion of the SGMs of the Kathmandu valley are living with 2 or more NCDs risk factors. Compared to the data from the general population reported in the 2019 STEPS survey, the prevalence of current smoking and harmful consumption of alcohol is higher among SGMs. These findings indicate a need to prioritize SGMs in NCDs-related to surveillance, policy planning, prevention, and control interventions.^33^

## Acknowledgments

Our sincere appreciation goes to individuals responding to the questionnaire and the organizations like Blue Diamond Society and the Federation for Sexual and Gender Minorities Nepal (FSGMN) for their constant communication, coordination, and support.

## Author contributions

**Conceptualization:** Bikram Poudel.

**Data curation:** Bikram Poudel, Kiran Paudel, Bikram Adhikari, Rajan Paudel, Sandesh Bhusal, Nabin Adhikari, Tara Ballav Adhikari.

**Formal analysis:** Bikram Poudel, Kiran Paudel.

**Investigation:** Bikram Poudel, Rajan Paudel.

**Methodology:** Bikram Poudel, Kiran Paudel, Bikram Adhikari, Rajan Paudel.

**Project administration:** Bikram Poudel.

**Resources:** Bikram Poudel.

**Software:** Bikram Poudel, Vishnu Prasad Sapkota.

**Supervision:** Vishnu Prasad Sapkota, Roman Shrestha.

**Validation:** Bikram Poudel, Vishnu Prasad Sapkota.

**Visualization:** Bikram Poudel.

**Writing—original draft:** Bikram Poudel, Kiran Paudel, Rajan Paudel, Sandesh Bhusal, Nabin Adhikari, Tara Ballav Adhikari, Vishnu Prasad Sapkota, Roman Shrestha.

**Writing—review & editing:** Bikram Poudel, Kiran Paudel, Bikram Adhikari, Rajan Paudel, Sandesh Bhusal, Nabin Adhikari, Tara Ballav Adhikari, Vishnu Prasad Sapkota, Roman Shrestha.
